# Identifying Key Factors Influencing Non-adherence to Imatinib Therapy in Patients With Chronic Myeloid Leukemia

**DOI:** 10.7759/cureus.79831

**Published:** 2025-02-28

**Authors:** Deepak Pandey, Preeta Chugh, Sumita Chaudhry

**Affiliations:** 1 Pharmacology, Vardhman Mahavir Medical College and Safdarjung Hospital, New Delhi, IND; 2 Internal Medicine, Vardhman Mahavir Medical College and Safdarjung Hospital, New Delhi, IND

**Keywords:** adherence, chemotherapy, myeloproliferative disorder, quality of life (qol), tyrosine kinase inhibitors (tki)

## Abstract

Background: The effective treatment of chronic myeloid leukemia (CML) remains a challenge due to patient non-adherence to imatinib. Medication non-adherence can lead to poor treatment outcomes, increased healthcare costs, and potential development of drug resistance in patients. In this study, we aimed to assess the factors associated with non-adherence to imatinib therapy in newly diagnosed CML patients.

Methods: In this study, 82 adult patients initiated on imatinib therapy were recruited. Adherence was measured using the medication possession ratio (MPR) and visual analogue scale (VAS) at 90 days. Demographic, clinical and quality-of-life variables were assessed at baseline. Pearson's correlation and linear regression analyses were performed to identify associations and independent predictors of non-adherence, respectively.

Results: A total of 22% (18/82) of patients were non-adherent (MPR ≤ 85). Self-reported adherence via VAS correlated significantly with MPR. Among various factors, cognitive function score and occurrence of an adverse drug reaction (ADR) were associated with adherence. The impact of disease on daily life score was negatively associated with adherence. The independent predictors of adherence were role function score and impact of disease on mood score.

Conclusion: This study identified cognitive and role function, impact of disease on daily life and mood and occurrence of ADR as variables influencing non-adherence to imatinib in CML patients. Early identification of these factors can guide interventions to improve adherence and treatment outcomes.

## Introduction

Imatinib has revolutionized the treatment of chronic myeloid leukemia (CML), achieving remission in approximately 80% of patients over the past two decades [1]. However, non-adherence to imatinib therapy remains a significant challenge, with studies reporting non-adherence rates ranging from 15% to 50% [2,3]. This non-adherence can lead to poor treatment outcomes, increased healthcare costs, and potential development of drug resistance [4].

Understanding the factors influencing adherence to imatinib is crucial for developing effective interventions to improve treatment success. Previous studies have explored various potential factors including demographic characteristics, disease-related parameters, and quality of life (QoL) aspects [5-7]. However, the independent predictive role of these factors remains unclear. This study aimed to address this gap by evaluating a comprehensive range of factors at the initiation of imatinib treatment and identifying their independent associations with non-adherence in newly diagnosed CML patients. This article was previously posted to the Authorea preprint server on June 14, 2024 [8].

## Materials and methods

Study design and participants

This study employed a prospective observational design. A total of 82 adult patients with chronic phase CML (confirmed by BCR-ABL transcripts) and initiated on imatinib (400 mg/day) who visited the hematology outpatient department of Vardhman Mahavir Medical College and Safdarjung Hospital, New Delhi (India), between December 2020 and January 2022 were included in the study. Written informed consent was obtained from all participants.

Data collection

Baseline data collection was done through a case record form and it included details of demographic parameters, namely, age, gender, smoking history, and alcohol intake. Clinical parameters, which included liver and spleen size, complete blood count, and BCR-ABL levels, were assessed. Disease risk assessment was done using prognostic scores, namely, Sokal score and EUTOS long-term survival (ELTS) score (EUTOS stands for European Treatment and Outcome Study for CML) [9,10]. The quality of life of patients was assessed using the European Organisation for Research and Treatment of Cancer Quality of Life Questionnaire Core 30 (EORTC QLQ-C30) and EORTC QLQ-CML24 [7,11]. The EORTC QLQ-C30 is an integrated system for assessing the health-related QoL of cancer patients participating in international clinical trials. It includes five functional scales, three symptom scales, a global health status/QoL scale, and six single items. The EORTC QLQ-CML24 is a supplementary 24-item questionnaire module to be employed in conjunction with the QLQ-C30 and is specially drafted for patients of CML. Both of these were administered by the interviewer in Hindi and English to assess the global health status, functional scales, and the impact of disease on various dimensions of quality of life. The completion rate of these questionnaires was 100%.

Adherence assessment was done using the “medication possession ratio” (MPR) that was calculated based on the self-reported medication intake every 15 days over a period of 90 days. At each 15-day interval, patients were asked to report any missing doses and the total number of days for which they took the medicine. Additionally, for subjective feedback on adherence, a visual analogue scale (VAS) was completed by patients, physicians, and a third person/caretaker. This included rating a patient's perceived adherence behavior on a scale of 0 to 10, where a score of 0 indicated complete non-adherence while a score of 10 implied complete adherence to treatment.

Toxicity assessment included recording any adverse drug reaction (ADR) reported by a patient during the 90-day period. This was initially recorded at each 15-day interval in the form of open-ended questions about the intake of medicines, well-being of the patient, and incidence of any ADR or symptom per se after the initiation of imatinib. This was followed by close-ended questions asking about specific adverse events related to imatinib.

Statistical analysis

Pearson's correlation analysis was used to evaluate associations between various factors and non-adherence (MPR ≤ 85). Multivariate linear regression analysis was performed to identify independent predictors of non-adherence.

## Results

Among the 82 patients, 18 patients (22%) were found to be non-adherent (MPR ≤ 85); the mean MPR for all patients was 93.5 ± 8.5 (out of 100). The subjective assessment of adherence as per VAS was converted into a score of 0 to 10 where “0” denoted no adherence at all and “10” denoting full adherence. The average scores were 9.78 ± 0.51, 9.01 ± 0.73 and 9.78 ± 0.51 by patients themselves, physician and third person/caretaker, respectively, in adherent patients. In non-adherent patients, the average scores by patients, physician and third person/caretaker were 7.01 ± 1.07, 6.55 ± 1.16 and 6.5 ± 1.21, respectively. The mean values of different variables in adherent and non-adherent patients are presented in Table 1.

**Table 1:** Factors influencing adherence to treatment EUTOS: European Treatment and Outcome Study for CML; ELTS score: EUTOS long-term survival score; EORTC QLQ-C30: European Organisation for Research and Treatment of Cancer Quality of Life Questionnaire Core 30; EORTC QLQ-CML24: 24-item EORCT Quality of Life Questionnaire for chronic myeloid leukemia

Variables	Adherent	Non-adherent
Demographic variables		
Age (years)	37.67 ± 12.6	32.8 ± 8
Male	38 (59.4%)	9 (50%)
Clinical parameters		
Liver size	4.9 ± 1.8	5.13 ± 1.9
Spleen size	5.2 ± 3.9	6.7 ± 4.5
Prognostic scores		
Sokal score	0.95 ± 0.3	0.98 ± 0.4
ELTS score	1.6 ± 0.5	1.58 ± 0.3
Scores derived from EORTC QLQ-C30		
Global health status score	59.6 ± 13.2	61.5 ± 18.5
Physical function score	81.1 ± 12.1	80.3 ± 17.2
Role function score	83.3 ± 15.1	76.9 ± 18.2
Emotional function score	88.9 ± 11.1	87.9 ± 14.1
Cognitive function score	98.4 ± 5.7	94.4 ± 14.1
Social function score	97.9 ± 6.9	98.1 ± 7.8
Scores derived from EORTC QLQ-CML24		
Impact of disease on mood score	17.1 ± 14.6	9.2 ± 15.1
Impact of disease on daily life score	4.1 ± 8.3	8.6 ± 5.5
Satisfaction with care score	90.6 ± 21.4	83.3 ± 30.2
Satisfaction with social life score	98.9 ± 5.8	100 ± 0

On statistical analysis using Pearson's correlation, the occurrence of an adverse drug event (r^2^ = 0.225, p = 0.042) displayed a significant positive correlation with adherence to treatment. The most commonly reported adverse effect of imatinib was nausea and vomiting (36.5%), followed by diarrhea (18.3%) and edema (14.6%), occurring mostly within the first two weeks. Skin rash (9.7%) and other effects like musculoskeletal pain, abdominal pain, and tenesmus were also reported. Among QoL parameters, the “cognitive function score” (derived from the EORTC QLQ-C30 questionnaire) had a significant positive correlation (r^2^ = 0.248, p = 0.025) with adherence while the “impact of disease on daily life score” (derived from the EORTC QLQ- CML24 questionnaire) had a significant negative correlation (r^2^ = -0.227, p = 0.041) with adherence.

On multivariate linear regression analysis, “role function score” (derived from the EORTC QLQ-C30 questionnaire) was found to be the predictor of adherence to treatment (unstandardized coefficient B = 0.155; standard error = 0.075; coefficient beta = 0.290; T = 2.072; p = 0.042). Also, the “impact of disease on mood score” (derived from the EORTC QLQ-CML24 questionnaire) was found to be independently predicting the adherence to treatment (unstandardized coefficient B = 0.208; standard error = 0.079; coefficient beta = 0.367; T = 2.628; p = 0.011).

Demographic variables and disease parameters did not significantly influence the adherence to treatment.

## Discussion

Non-adherence to imatinib remains a significant challenge in the management of CML leading to poor outcomes and increased healthcare costs [2,12]. We observed that the presence of an ADR affected adherence to treatment. The values of cognitive function score, role function score, impact of disease on daily life score and impact of disease on mood score values also impacted adherence to treatment.

Interestingly, the presence of an ADR was positively associated with adherence in our study. This suggests that patients experiencing ADRs might be more mindful of taking their medication, possibly due to heightened awareness of their illness and treatment regimen. However, this contrasts with previous findings from a European study, which reported no significant association between ADRs and adherence [3].

It is known that cognitive function plays a crucial role in medication adherence, particularly in patients who manage their medications independently [4]. In this study, the cognitive function score indicates the level of functionality in the cognitive dimension, e.g., the ability to focus on activities like watching television and to remember trivial things. A direct association with adherence reaffirms the role of cognitive functioning in adhering to medications. The role function score indicates the ability of a person to do his/her daily work or to pursue leisure activities. A good role function score indicates better adherence implying that those individuals who were not limited in doing daily activities were more likely to take medicines as directed. The previous literature has demonstrated the negative impact of functional limitations on adherence behavior [13]. In a similar way, the impact of disease on daily life score predicted non-adherence to treatment. It showed that the patients who felt the burden of treatment or required social support to cope with disease were more likely to not take medicines as prescribed. This is consistent with studies conducted in the USA and Europe, where a higher treatment burden was linked to non-adherent behavior [14,15].

Furthermore, the independent association of the impact of disease on mood score with adherence indicates that patients who were worried or concerned about disease treatment had better adherence to treatment. This highlights the potential role of healthcare-seeking behavior and self-care practices in promoting adherence. Our results corroborate findings of Kapoor et al., who reported a positive association between depressive symptoms and adherence [5].

It is noteworthy that we did not observe significant associations between demographic or clinical characteristics and adherence. Variable results have been observed regarding the influence of age on adherence in previous studies. The ADAGIO (Adherence Assessment With Glivec: Indicators and Outcomes) study revealed a negative association of age with adherence [2]. In another study, Marin et al. observed a positive association between age and adherence [3,4]. This difference may be attributed to methodological variations as we included only adult patients in our study. Darkow et al. demonstrated that male patients had better adherence while the ADAGIO study demonstrated that female patients had better adherence to treatment [2,16]. Our study did not find any association of adherence with gender. Previous studies from India have also not reported any influence of gender on adherence [7]. Our study found no significant link between smoking or alcohol intake and imatinib adherence in CML patients, unlike previous studies suggesting that unhealthy lifestyle behaviors may reduce medication adherence. This discrepancy may be due to differences in patient populations and disease contexts.

Also, we did not observe any impact of prognostic scores, clinical status and molecular markers on adherence to treatment. This is in contrast to findings of Marin et al. and Guerin et al. who demonstrated increased non-adherence in patients with a sub-optimal clinical response [3,17]. This may be due to the fact that these were retrospective analyses and did not account for the temporal relationship between treatment response and adherence. Also, this could be attributed to methodological variations, sample characteristics, and healthcare system factors influencing adherence behaviors across different settings [18].

This study had a few limitations that should be considered when interpreting the findings. Patients were followed up for only 90 days, a time frame that may not be sufficient to capture long-term adherence patterns and their impact on treatment outcomes. A key limitation of our study is the exclusion of economic factors that have been shown to influence adherence to imatinib in a previous research [19]. Since imatinib was provided free of cost at our hospital, economic constraints were not a direct concern. In addition, categorizing individuals into different economic strata was challenging due to their diverse backgrounds and variations in economic benchmarks in rural and urban settings.

## Conclusions

In conclusion, we identified various factors influencing adherence to imatinib treatment. The presence of an ADR and the level of cognitive functioning of newly diagnosed CML patients correlated with their adherence to imatinib therapy. In contrast, a higher perceived treatment burden and need for social support were associated with non-adherence. Also, the role function score and impact of disease on mood score were found out to be independent predictors of their adherence behavior. Our findings highlight the importance of considering patients' functional limitations, disease burden, and concern towards disease treatment in assessing the adherence to treatment. These factors can be identified at the initiation of therapy through simple subjective assessments, and tools for improving adherence can be used for better treatment outcomes. Future research with longer follow-up periods and control groups, considering the potential influence of external factors, would be beneficial to strengthen the generalizability and robustness of study findings.
